# Amyloid, tau, and astrocyte pathology in autosomal-dominant Alzheimer’s disease variants: *AβPP*arc and *PSEN1*D*E9*

**DOI:** 10.1038/s41380-020-0817-2

**Published:** 2020-06-25

**Authors:** Laetitia Lemoine, Per-Göran Gillberg, Nenad Bogdanovic, Inger Nennesmo, Laure Saint-Aubert, Matti Viitanen, Caroline Graff, Martin Ingelsson, Agneta Nordberg

**Affiliations:** 1 Division of Clinical Geriatrics, Department of Neurobiology, Care Sciences and Society, Center for Alzheimer Research, Karolinska Institutet, Stockholm, Sweden; 2 Theme Aging, The Aging Brain, Karolinska University Hospital, Stockholm, Sweden; 3 Department of Pathology, Karolinska University Hospital, Stockholm, Sweden; 4 ToNIC, Toulouse NeuroImaging Center, University of Toulouse, Inserm, UPS, Toulouse, France; 5 Nuclear Medicine Department, University Hospital of Toulouse, Toulouse, France; 6 Department of Geriatrics, Turku City Hospital, Turku University, Turku, Finland; 7 Division of Neurogeriatrics, Department of Neurobiology, Care Sciences and Society, Center for Alzheimer Research, Karolinska Institutet, Stockholm, Sweden; 8 Theme Aging, Unit for Hereditary Dementia, Karolinska University Hospital, Stockholm, Sweden; 9 Department of Public Health and Caring Sciences, Geriatrics, Uppsala University, Uppsala, Sweden

**Keywords:** Diagnostic markers, Neuroscience

## Abstract

Autosomal-dominant Alzheimer’s disease (ADAD) may be associated with atypical amyloid beta deposits in the brain. In vivo amyloid imaging using ^11^C-Pittsburgh compound B (PiB) tracer has shown differences in binding between brains from ADAD and sporadic Alzheimer’s disease (sAD) patients. To gain further insight into the various pathological characteristics of these genetic variants, we performed large frozen hemisphere autoradiography and brain homogenate binding assays with ^3^H-PiB, ^3^H-MK6240-^3^H-THK5117, and ^3^H-deprenyl for detection of amyloid fibrils, tau depositions, and activated astrocytes, respectively, in two *AβPParc* mutation carriers, one *PSEN1ΔE9* mutation carrier, and three sAD cases. The results were compared with Abeta 40, Abeta 42, AT8, and GFAP immunostaining, respectively, as well as with Congo red and Bielschowsky. PiB showed a very low binding in *AβPParc*. A high binding was observed in *PSEN1ΔE9* and in sAD tissues but with different binding patterns. Comparable ^3^H-THK5117 and ^3^H-deprenyl brain homogenate binding was observed for *AβPParc*, *PSEN1ΔE9*, and sAD, respectively. Some differences were observed between ^3^H-MK6240 and ^3^H-THK5117 in ADAD. A positive correlation between ^3^H-deprenyl and ^3^H-THK5117 binding was observed in *AβPParc*, while no such correlation was found in *PSEN1ΔE9* and sAD. Our study demonstrates differences in the properties of the amyloid plaques between two genetic variants of AD and sAD. Despite the lack of measurable amyloid fibrils by PiB in the *AβPParc* cases, high regional tau and astrocyte binding was observed. The lack of correlation between ^3^H-deprenyl and ^3^H-THK5117 binding in *PSEN1ΔE9* and sAD in contrast of the positive correlation observed in the *AβPParc* cases suggest differences in the pathological cascade between variants of AD that warrant further exploration in vivo.

## Introduction

In a small percentage of patients, Alzheimer’s disease (AD) is characterized by an early onset due to a mutation in one of three identified genes: amyloid-beta precursor protein (*AβPP*), Presenilin 1 (*PSEN1*), and Presenilin 2 (*PSEN2*). Mutation-specific features have been described, both in vitro and in vivo, showing a faster progression of the disease in Autosomal-dominant Alzheimer’s disease (ADAD) variants in comparison to the sporadic form of Alzheimer’s disease (sAD) [1]. However, not all identified mutations will result in the clinical phenotype of AD [2].

In *PSEN1*D*E9* brains, (due to an exon 9 deletion in *PSEN1* (*PSEN1*D*E9*) identified in the FINN2 family [3]) accumulation of large plaques composed of amyloid beta 42 and amyloid beta 40 without a compact amyloid core so called ‘cotton wool’ plaques have been reported [4]. Significant difference was observed between *PSEN1*D*E9* mutation and sAD with significantly higher Abeta 42/40 ratio in *PSEN1*D*E9* [4]. *PSEN1*D*E9* is distinct from the sAD phenotype and could also result for some cases, in a clinical phenotype that includes spastic paraparesis [3]. The *AβPParc* mutation (p. E693G) is pathologically characterized by the presence of ring-shaped amyloid plaques without amyloid core [5]. Those plaques are Congo red negative but amyloid beta 42-positive on the ring as observed using amyloid beta 1–42 antibodies [1, 6]. The clinical phenotype in *AβPParc* is similar to that in patients with sAD but with an earlier onset of the disease (45–57 years) [3]. Previous in vitro studies have shown increased levels of oligomeric and protofibrillar forms of amyloid beta in *AβPParc* oligomeric preparations [7, 8]. Philipson et al., in 2012 compared both the amyloid plaques structure and the accumulations of N- and C-truncated *Aβ* in *AβPParc, PSEN1*D*E9 and sAD*, and they could observe differences in length accumulation of N- and C-truncated Aβ40 and 42 between the two mutation and sAD in parenchymal plaques as well as in cerebral amyloid angyopathy [9]. The in vivo PET binding of ^11^C-Pittsburgh compound B (PiB) is low in both symptomatic and nonsymptomatic *AβPParc* carriers in comparison to patients with sAD while levels of amyloid beta 42, total tau and P-tau in the cerebrospinal fluid and cerebral metabolism as measured by ^18^F-fluorodeoxyglucose (FDG) PET are comparable with sAD [6, 10, 11].

In the *PSEN1*D*E9* mutation carriers, ^11^C-PIB PET has demonstrated increased amyloid plaque deposition in comparison to control and with a distinct pattern than sAD with a higher ^11^C-PIB binding in the putamen in comparison to sAD patients [12]. ^18^F-FDG hypometabolism and pathological levels of CSF biomarkers were comparable to those observed in sAD patients [12].

In this study, our aim was to further characterize the neuropathological features of two *AβPParc* and one *PSEN1*D*E9* mutation carrier using autoradiography with PET tracers as well as immunohistochemistry, in order to assess the extent and regional distribution of plaques, tangles, and activated astrocytes. An ante-mortem/postmortem binding comparison was performed for one *AβPParc* case.

## Material and methods

### Autopsy material

Large frozen whole left hemisphere sections were obtained from two *AβPParc* (*AβPParc1*, provided by the Brain Bank at Karolinska Institutet; *AβPParc2*, provided by the Uppsala University brain bank), one *PSEN1*D*E9* (provided by the Department of Pathology, University of Helsinki, Helsinki, Finland) and from the right hemisphere for three sAD brains (provided by the Neuropathology of Dementia Laboratory, Indiana University School of Medicine, Indianapolis, IN, USA). Direct comparison between the cases should be taken with caution due to the fact that large frozen hemisphere sections are rare material and the sections were not from the exact same coronal anatomical level. For the two *AβPParc*, and the three sAD pieces of frontal, temporal, and entorhinal cortices, as well as hippocampus and caudate nucleus were dissected and used for binding assay studies. For the *PSEN1*D*E9*, frontal, temporal cortices, and caudate were available. Frozen homogenates of frontal cortex, temporal cortex, hippocampus, and caudate nucleus from two nondemented controls (obtained from the Netherlands Brain Bank) were used for binding assays. The demographic data are presented in Table 1. Clinical description of the patients with *AβPParc* and *PSEN1ΔE9* mutations is available on Supplementary data 1.

**Table 1:** Table representing the clinical information.

	Sex	Age of onset (years)	Age at death (years)	Braak stage	APOE	PMI (h)
*AβPParc1*	F	53	66	VI	3/3	>30
*AβPParc2*	M	61	64	VI	3/3	12
*PSEN1ΔE9*	F	51	66	N/A	3/3	5
AD1	F		59	VI	3/3	4
AD2	F		73	V	3/3	1.5
AD3	F		59	V	3/4	10
Control 1	M		62	I	3/3	7
Control 2	F		71	I	3/2	7
Control 3	M		79	II	3/3	9

Demographic information for patients with autosomal-dominant Alzheimer’s disease, patients with sporadic Alzheimer’s disease and normal controls.

*AD* Alzheimer’s disease, *APOE* apolipoprotein E, *AβPParc* *Arctic amyloid-β protein precursor* mutation, *F* female, *M* male, *N/A* not applicable, *PMI* postmortem interval, *PSEN1ΔE9* *PS1* exon 9 deletion.

### Chemicals

^3^H-THK5117 and unlabeled THK5117 were synthesized by Novandi chemistry AB (Södertälje, Sweden; specific activity (SA) = 75 Ci/mmol). ^3^H-MK6240 and unlabeled MK6240 were synthesized by Merck & Co; SA: 44 Ci/mmol. ^3^H-PIB was custom synthesized by Novandi (Södertälje, Sweden; SA = 73 Ci/mmol). ^3^H-l-deprenyl was custom synthesized by Quotient Bioresearch (Cardiff, UK; SA = 85 Ci/mmol). Unlabeled (R)-(−)-deprenyl was purchased from Tocris Bioscience and BTA-1 was purchased from Sigma-Aldrich.

### Autoradiography

Autoradiography from all cases was carried out at room temperature (RT) on frozen coronal sections (100 μm thick using a Cryomacrotome Leica CM3600XP, Leica Biosystems, USA) after allowing them to dry. For ^3^H-PIB, the sections were preincubated for 15 min with Phosphate Buffer Saline (PBS) + 1% BSA, then incubated for 45 min with ^3^H-PIB (1 nM) in PBS + 0.1% BSA. Nonspecific (NSP) binding was determined with 1 µM BTA-1. For ^3^H-THK5117 and ^3^H-MK6240, the sections were preincubated 15 min with PBS + 0.1% BSA, then incubated for 1 h with ^3^H-THK5117 (3 nM) or ^3^H-MK6240 (1 nM) with the same buffer. NSP was determined with 1 µM unlabeled THK5117 or MK6240, respectively. Finally, for ^3^H-deprenyl autoradiography the sections were incubated with ^3^H-deprenyl (10 nM) for 1 h in Na–K phosphate buffer. NSP was determined with 1 µM of unlabeled deprenyl.

For all the radioligands, the binding reaction was terminated by washing 3 × 5 min with cold binding buffer (4 °C) followed by one dip in cold distilled water (4 °C). The sections were then dried and apposed on photostimulable phosphor-plates for 4 days for ^3^H-deprenyl and ^3^H-THK5117 and 7 days for ^3^H-PIB and ^3^H-MK6240. The photostimulable phosphor-plates were then read using a BAS-2500 imager and the results were analyzed using multigauge software to draw the regions of interest manually.

### Immunohistochemistry for neuropathological evaluation

Immunohistochemistry for neuropathological evaluation was performed on small paraffin-embedded sections from right brain hemisphere obtained from the same cases mentioned above (*AβPParc1, AβPParc2*, and *PSEN1*D*E9)* (For *AβPParc1* 6 μm thick sections on no coated slides were used; for *AβPParc2* 7 μm thick sections on coated slides were used and for *PSEN1*D*E9* 7 μm thick sections on superfrost slides were used).

A routine deparaffinization protocol was used. AT8 (Phospho-Tau, Ser202, Thr205) monoclonal antibody from Thermofisher, amyloid beta 1–42 (antiamyloid β42 antibody, clone G2-11 from Merck Millipore), and amyloid beta 1–40 (antiamyloid β40 antibody, clone G2-10 from Merck milipore) antibodies were used as follows: AT8 dilution 1:2500 stained in Roche Ventana immunostainer; amyloid beta 1–42 dilution 1:750 and amyloid beta 1–40 dilution 1:500 stained in Roche Ventana immunostainer but with 10 min in formic acid first after deparaffination.

### In vitro brain regional binding studies

Regional binding studies were carried out on fresh frozen tissues from frontal, temporal, and entorhinal cortices, caudate nucleus and hippocampus using ^3^H-PIB, ^3^H-deprenyl, and ^3^H-THK5117. Each compound was incubated with the brain homogenates and filtered after a tracer-specific incubation time, as follow: ^3^H-PIB (1 nM) was incubated in PBS for 2 h at RT with 0.1 mg/ml of tissue. NSP was determined using 1 µM of BTA-1. ^3^H-deprenyl (10 nM) was incubated for 1 h at 37 °C with 0.2 mg/ml of tissue using Na–K phosphate buffer. NSP was determined using 1 µM unlabeled deprenyl. ^3^H-THK5117 (3 nM) was incubated for 2 h at RT with 0.2 mg/ml of tissue using PBS + 0.1% BSA. NSP was determined using 1 µM unlabeled THK5117.

All experiments were terminated by filtering through glass fiber filter paper presoaked with polyethylenimine 0.3%, rinsing three times with cold binding buffer and then counting on a Beckman scintillation counter. Each experiment was performed at least in triplicate and analyzed using graph pad prism software.

### *AβPParc* in vivo imaging

Two years before death, the *AβPParc1* patient underwent a structural T1 MPRAGE MRI on a 3T (Siemens Trio) scanner at the Karolinska University Hospital, Huddinge (Sweden), and PET examinations with ^11^C-deprenyl, ^11^C-PIB, and ^18^F-FDG at the Uppsala PET Centre, Uppsala University (Sweden), on an ECAT EXACT HR + (Siemens/CTI) PET/CT scanner. Productions of the tracers and image acquisitions have been previously described [6].

## Results

### Autoradiography and immunostaining comparison

**Fig. 1:**
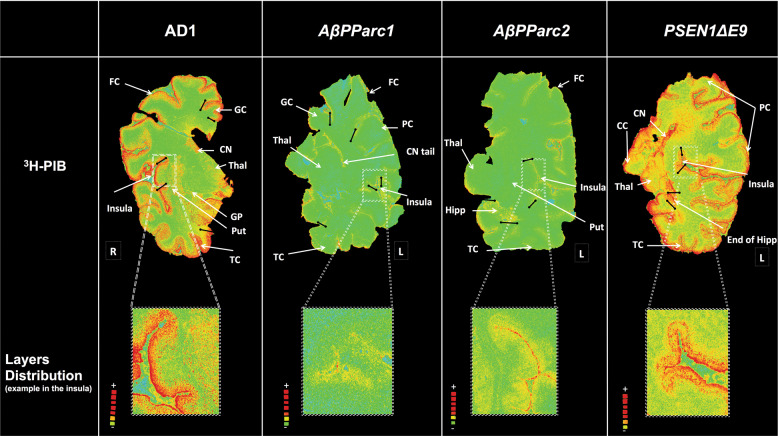
Representation of total binding autoradiography on large frozen sections with ^3^H-Pittsburg compound B. Autoradiography on large frozen sections from one Alzheimer’s disease brain (AD1), two *AβPParc* brains and one *PSEN1*D*E9* deletion brain. The top panel shows ^3^H-Pittsburgh compound B (PIB) autoradiography (standard: + = 4700 fmol/mg, − = 50 fmol/mg) and the bottom panel shows enlargements of the insula region to illustrate distribution in the layers. All images were put on the same threshold (47,545) from the raw images (16 bits: 0–65,535 (color scale)) for comparison. CN caudate nucleus, CC corpus callosum, FC frontal cortex, GC gyrus cingulate, GP globus pallidus, Hipp hippocampus, Put putamen, PC parietal cortex, TC temporal cortex, Thal thalamus.

**Fig. 2:**
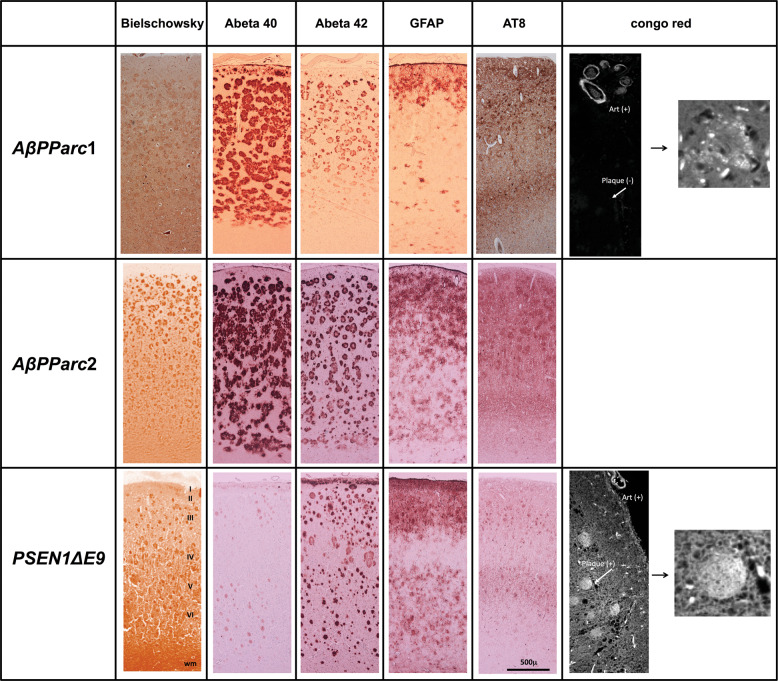
Immunostaining of *AβPParc1, AβPParc2*, and *PSEN1*D*E9* brain tissue. From left to right, Bielschowsky, Abeta 40, Abeta 42, glial fibrillary acidic protein (GFAP), and AT8 stains and Congo red.

The regional binding of ^3^H-PIB on large frozen brain sections of the two *AβPParc* mutation carriers, the *PSEN1*D*E9* mutation carrier and one sAD case is presented in Fig. 1. ^3^H-PIB binding was very low in the whole brain hemisphere sections of the two *AβPParc* brains in comparison to the sAD brain. In contrast, the binding in the *PSEN1*D*E9* brain was higher and comparable to that in the sAD brain, but with a more uneven distribution (‘cloudy’ pattern) (see enlargement in Fig. 1). Some ^3^H-PIB binding could also observed in the white matter of the *PSEN1*D*E9* brain. A comparison of the immunostaining using amyloid *β* 40, amyloid *β* 42, and Bielschowsky staining is presented in Fig. 2. The Bielschowsky stain bound to senile plaques and neurofibrillary tangles more intensely in the two *AβPParc* brains than in the *PSEN1*D*E9* brain. The amyloid *β* 40 and 42 staining was also intense in all layers for the two *AβPParc* brains, with more spreading in the upper layers. In the *PSEN1*D*E9* brain, amyloid *β* 42 staining was more intense than amyloid *β* 40 staining; amyloid staining was especially intense in layer 1 (corresponding to subpial amyloid accumulation). Amyloid beta 42 antibodies resulted in more compact, filled-in ‘cotton wool’ plaques in the *PSEN1*D*E9* brain than the ring-shaped plaques in the two *AβPParc* brains (Fig. 2). In the *AβPParc* brains, the specific ring shape of the plaques was observed predominantly with amyloid *β*42 staining, which was located on the outer rims of the plaques; amyloid *β* 40 binding was homogeneously distributed throughout the plaque formations. The prominent difference between the ‘cotton wool’ plaques in the *PSEN1*D*E9* brain and the ring-shaped plaques in the *AβPParc* brains was clearly illustrated by Congo red (see Fig. 2).

**Fig. 3:**
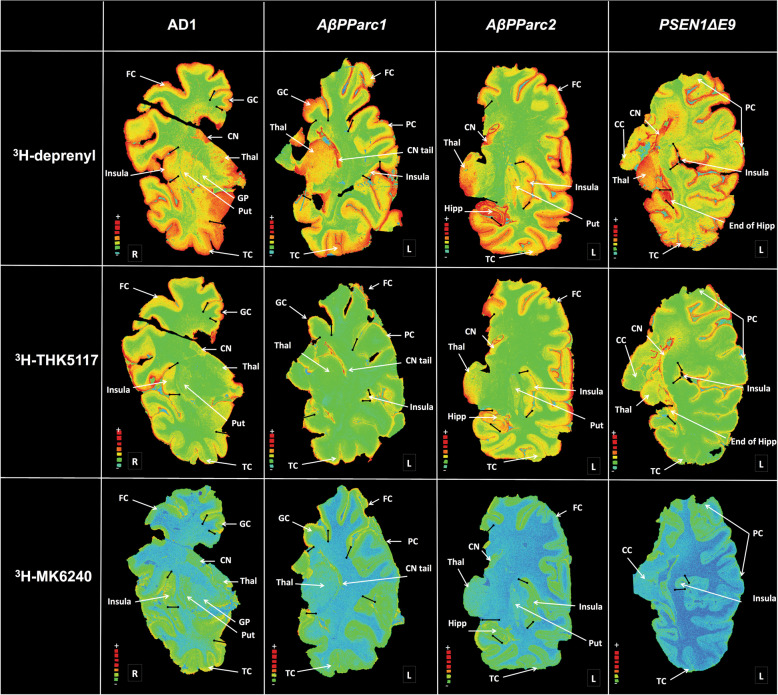
Representation of total binding autoradiography on large frozen sections with ^3^H-deprenyl, ^3^H-THK5117, and ^3^H-MK6240. Autoradiography on large frozen sections from one Alzheimer’s disease (AD) brain, two *AβPParc* brains and one *PSEN1*D*E9* deletion brain. The top panel shows ^3^H-deprenyl autoradiography (Standard: + = 4100 fmol/mg, − = 50 fmol/mg), middle panel shows ^3^H-THK5117 autoradiography (standard: + = 4500 fmol/mg, − = 30 fmol/mg) and the bottom panel shows ^3^H-MK6240 autoradiography (standard: + = 6500 fmol/mg, − = 70 fmol/mg). All images from deprenyl and THK5117 tracers were put on the same threshold (50,372) from the raw images (16 bits :0–65,535 (color scale)) for comparison. For MK6240 threshold were putted at 46,517 to allow comparison with other tracers. CN caudate nucleus, CC corpus callosum, FC frontal cortex, GC gyrus cingulate, GP globus pallidus, Hipp hippocampus, Put putamen, PC Parietal cortex, TC temporal cortex, Thal thalamus.

The regional binding pattern of ^3^H-deprenyl, ^3^H-THK5117, and ^3^H-MK6240 are presented in the Fig. 3. ^3^H-deprenyl autoradiography showed the most intensity of binding compared with the two-tau tracers using the same color scale. An interesting difference between the two genetic variants and sAD was the binding of ^3^H-deprenyl in the white matter of the *PSEN1*D*E9* as confirmed by intensive immunostaining using the glial fibrillary acidic protein (GFAP) stain (Fig. 2). While comparing the two-tau tracers ^3^H-THK5117 and ^3^H-MK6240, lower general binding intensity was observed for ^3^H-MK6240 due to different SA (44 Ci/mmol for ^3^H-MK6240 and 85 Ci/mmol for ^3^H-THK5117) as well as incubation concentration (1 nM for ^3^H-MK6240 and 4 nM for ^3^H-THK5117). Regional distribution binding was similar for the two-tau tracers in the sAD. For *AβPParc1*, ^3^H-MK6240 binding is higher than the one of ^3^H-THK5117. For *AβPParc2* and *PSEN1*D*E9* differences were observed between the two-tau tracers. Indeed, in *PSEN1*D*E9* lower ^3^H-MK6240 binding was observed in temporal area in comparison to other cases. ^3^H-THK5117 binding was more extensive throughout the cortical ribbon in the *AβPParc*2 brain than in the *AβPParc*1 brain, again confirmed by GFAP and AT8 immunostaining (Fig. 2). GFAP and AT8 immunostaining also showed differential bilayer distribution in the superficial and deep pyramidal layers of the three ADAD (data not shown). In the *PSEN1*D*E9* brain, AT8 staining was similar to that in the sAD brain but was less intense than that in the two *AβPParc* brains. In the *AβPParc*2 brain, GFAP and AT8 immunostaining was more intense throughout all the layers, including the superficial layer, than in the *AβPParc*1 brain. In both the *AβPParc* brains, AT8 staining followed the distribution of the amyloid plaques, depicting binding in the neuritic plaques. GFAP staining was more intense in the *PSEN1*D*E9* brain than in the two *AβPParc* brains, with intense binding in the upper layers (including the molecular layer with subpical positivity) and in the white matter.

### Quantitative assessment of the regional binding distribution of ^3^H-PIB, ^3^H-THK5117, and ^3^H-deprenyl

**Fig. 4:**
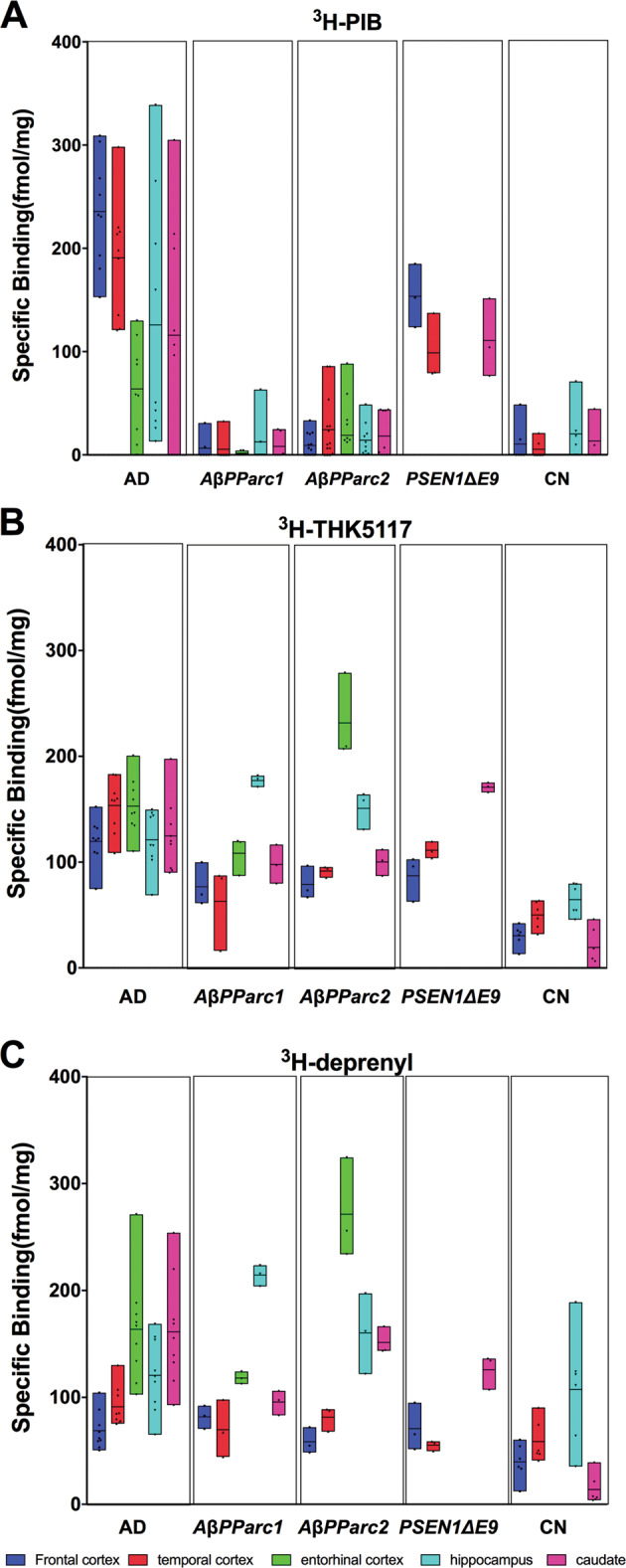
Regional-specific binding distribution assays. Regional-specific binding distribution assays using single concentrations of (**a**) ^3^H-Pittsburgh compound B (PIB); (**b**) ^3^H-THK5117 and (**c**) ^3^H-deprenyl in two *AβPParc* brains, one *PSEN1*D*E9* brain, three sporadic Alzheimer’s disease (AD) brains and three normal control brains. Frontal cortex, entorhinal cortex, caudate nucleus, temporal cortex, and hippocampus were investigated depending on the availability of the tissue.

Regional binding of the three PET tracers was quantitatively assessed using brain homogenates in binding assays; results are shown in Fig. 4. Low ^3^H-PIB binding was observed in the two *AβPParc* brains compared with intermediate binding in the *PSEN1*D*E9* brain and high binding in sAD (Fig. 4a). The binding in the caudate was in general similar to the cortical binding. In the AD cases the caudate showed an intermediate binding while both higher and lower cortical binding were observed, probably due to larger intra-cases variability. Higher ^3^H-THK5117 binding was detected in the frontal and temporal cortices in the sAD brains compared with the *AβPParc* and *PSEN1*D*E9* brains (Fig. 4b). The highest ^3^H-THK5117 mean binding was observed in the hippocampus of the *AβPParc*1 brain, in the entorhinal cortex of the *AβPParc*2 brain, and in the caudate nucleus of the *PSEN1*D*E9* brain. Binding in the frontal and temporal cortices was similar for the two *AβPParc* and slightly higher in the *PSEN1*D*E9* brains. The binding in caudate is higher than in the cortex in all mutation brains except in sAD and control.

^3^H-deprenyl binding was similar in the frontal cortex and in the caudate nucleus for both sporadic and mutation cases. Greater ^3^H-deprenyl binding was observed in the hippocampus of the *AβPParc1* case and the entorhinal cortex of *AβPParc*2 compared with sAD cases (Fig. 4c). Caudate binding is higher than the cortex binding in all AD brains while lower than the entorhinal cortex and hippocampus in the two *AβPParc*.

### Relationship between ^3^H-deprenyl and ^3^H-THK5117 regional binding

**Fig. 5:**
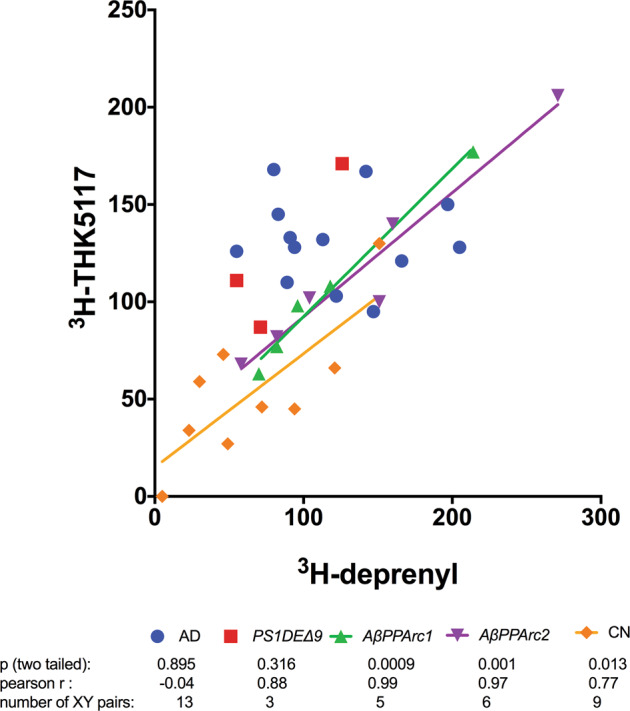
Correlation between ^3^H-THK5117 and ^3^H-deprenyl binding. Comparison of deprenyl and THK5117 regional binding distributions in two *AβPParc* brains, one *PSEN1*D*E9* brain, three Alzheimer’s disease brains, and three normal control brains. Frontal cortex, temporal cortex, entorhinal cortex, caudate nucleus, and hippocampus tissue was used depending on availability. *n* = number of samples investigated.

A significant positive correlation between ^3^H-deprenyl and ^3^H-THK5117 binding was observed for the two *AβPParc* in different brain regions, while no such significant correlations were observed for the sAD or *PSEN1*D*E9* brains (Fig. 5). No significant correlations were seen between ^3^H-PIB and ^3^H-THK5117 in any group (Supplementary data 2a). Between ^3^H-PIB and ^3^H-Deprenyl significant negative correlation could be observe only in sAD (Supplementary data 2b).

### *AβPParc*1 brain multitracer PET scan

**Fig. 6:**
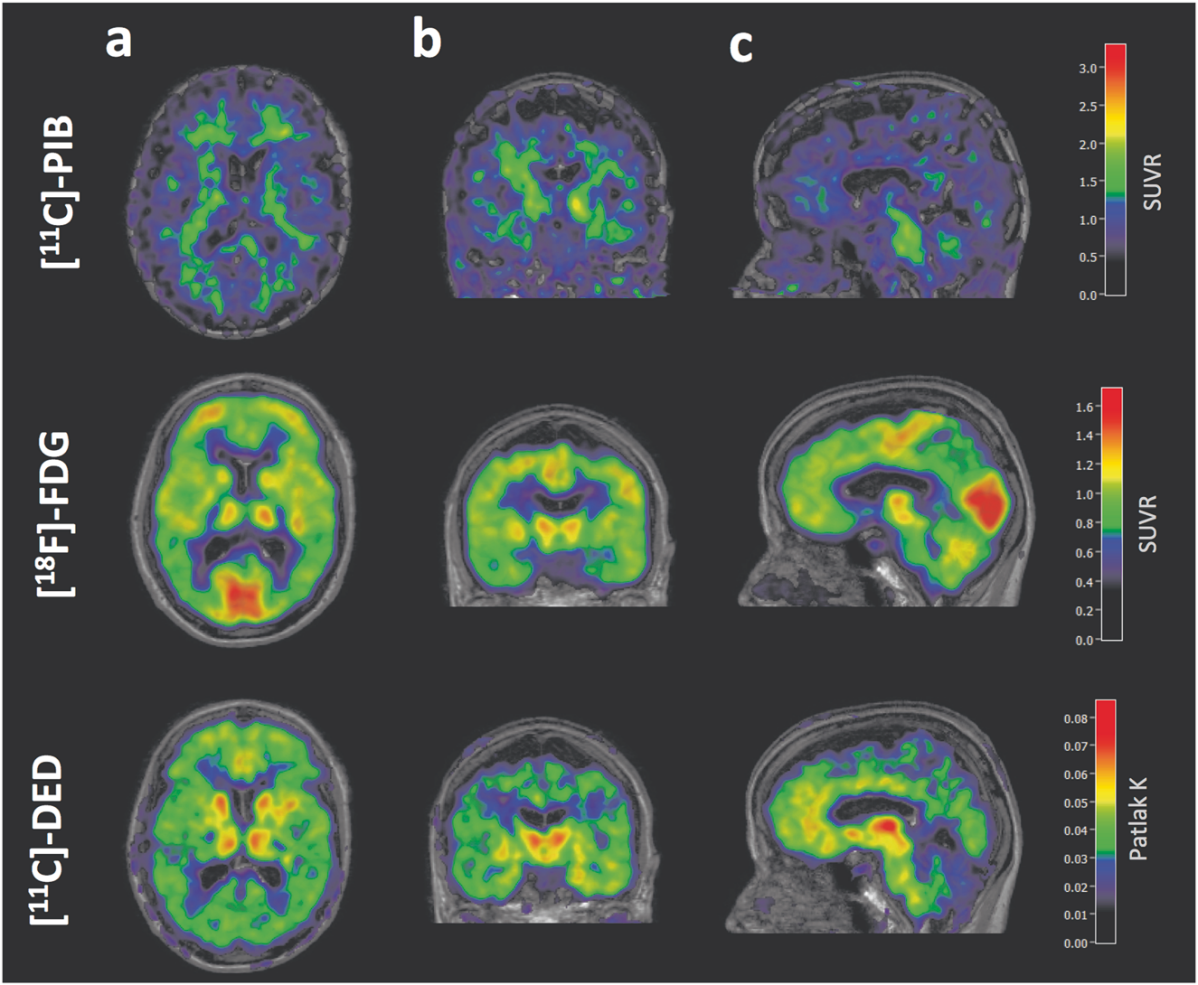
Multitracer PET scans for *AβPParc1* mutation carrier Multitracer PET scan for an *AβPParc* mutation carrier (*AβPParc1*) using ^11^C-Pittsburgh compound B (PIB), ^18^F-fludeoxyglucose (FDG), and ^11^C-deprenyl tracers. (^11^C-PIB and ^18^F-FDG late sum images (40–60 and 30–45 min, respectively) were created and co-registered onto the T1 MRI image. The whole pons was used as a reference for both tracers as it was found to be preserved from pathology in both autosomal-dominant and sporadic Alzheimer’s disease [14]. The ^11^C-deprenyl dynamic PET images were co-registered onto the T1 MRI image, and a modified reference Patlak model [15] was applied to the 20–60 min image using PMOD software, with the cerebellum as the modified reference region [16] to generate individual 3D parametric Patlak slope images (unit = min^−1^)). **a** horizontal section; **b** coronal section; **c** sagittal section.

Parametric images of PET acquisitions using ^11^C-L-deprenyl, ^18^F-FDG, and ^11^C-PIB in the *AβPParc1* carrier performed 2 years prior to death are shown in Fig. 6. The ^11^C-PIB PET scan showed very low binding and considered to be amyloid negative according to used cutoff values [13]. The ^18^F-FDG PET scan showed cortical hypometabolism, predominantly in the temporo-parietal regions, and ^11^C-deprenyl showed high binding in the cortical regions and basal ganglia (Fig. 6) (see Supplementary data 3 for in vivo–in vitro correlation for *AβPParc*1).

## Discussion

The aim of the study was to compare the neuropathological features of the *AβPParc* and *PSEN1*D*E9* mutations with those of sAD using in vitro binding studies of autopsied brain tissue with PET ligands, in order to be able to understand the relationships between the different hallmarks of AD pathology.

Autoradiographies with ^3^H-PIB binding confirmed the in vivo data: the lack of ^3^H-PIB binding in the two *AβPParc* brains reflects the absence of fibrillar form (Congo positive) amyloid plaques in the two *AβPParc* brains. However, ^3^H-PIB autoradiography showed relatively intense binding in the *PSEN1*D*E9* brain, comparable to sAD brains. ^3^H-PIB binding in the *PSEN1*D*E9* brain was distributed mostly in the upper layers of the cortex, with specific binding also detected in the white matter. The *PSEN1*D*E9* brain has been shown to have rich amyloid angiopathy as observed with thioflavin-S [3] as well as *AβPParc* [1]. Interestingly, we confirmed these differences in the ^3^H-PIB binding pattern using amyloid *β* 42 and 40 antibody immunostaining. A striking difference between *AβPParc* and *PSEN1*D*E9* was observed with the amyloid *β* 40 antibody; there was much less staining in the *PSEN1*D*E9* brain than in the two *AβPParc* brains. This major difference illustrates the different biological compositions of the amyloid plaques in *AβPParc* and *PSEN1*D*E9* mutations. These differences were confirmed with Congo red staining; there was a total Congo red negative core for the *AβPParc* brains and some positivity for the *PSEN1*D*E9* brain, but it was still more diffusely and evenly distributed (like cotton wool) in comparison to the sAD brains. In previous studies, Philipson et al. [9] have also demonstrated the differences between the biological composition of the amyloid plaques of *AβPParc* and *PSEN1*D*E9* mutations. Moreover, Verkkoniemi et al., showed that both noncored, cored as well as diffuse plaques were found in the cerebral cortex of a patient with the FINN2 *PSEN1*D*E9* mutation [14].

The other characteristic pathological hallmarks of AD, such as tau deposits and activated astrocytes, measured by ^3^H-THK5117/^3^H-MK6240 and ^3^H-deprenyl, respectively, showed more similarities across the *AβPParc*, *PSEN1*D*E9* and less similar with sAD brains. The most striking similarities is that both tau deposits and activated astrocytes binding are the highest in the limbic areas > caudate > cortical areas for the two *AβPParc* and *PSEN1*D*E9*, when in the sAD, these differences are not pronounced.

For both the *PSEN1*D*E9* and the *AβPParc* mutations, similar laminar distributions throughout the cortical ribbon were observed for ^3^H-THK5117 and ^3^H-deprenyl. The similar results have already been described for sAD autoradiography using ^3^H-THK5117 and ^3^H-deprenyl [15]. This bilayer pattern was confirmed using GFAP and AT8 immunostaining. Similarly, the activated astrocytes and tau deposits seemed to both describe a bilayer distribution for both mutations. Interestingly, a positive correlation between ^3^H-deprenyl and ^3^H-THK5117 binding was only observed in the two *AβPParc* and the control brains, while there was no significant correlation in either the *PSEN1*D*E9* or the sAD brains. These observations suggest that it might be a closer relationship between activated astrocytes and tau deposits in *AβPParc* brains than in *PSEN1*D*E9* brains. Since some off-target binding to monoamine oxidase B (MAO-B) has been reported for the first generation tau tracer, THK5117, we also included MK6240, a tau tracer from the second generation, with no MAO-B off-target binding [16]. No regional differences in regional binding pattern were observed between the two-tau tracers in sAD except for a difference in intensity of binding that can be due both to the difference in specific radioactivity between the two tracers as well as probably more NSP binding for THK5117. However, some differences were observed between the two-tau PET tracers since ^3^H-MK6240 showed lower binding in the *PSEN1DE9* and *AβPParc2* variants in comparison to *AβPParc1* cases and the sAD cases. This observation might suggest some difference in binding properties between these two-tau PET tracers at least in AD mutation cases. It will be interesting to study further the underlying cause of the difference between the bindings of the two-tau PET tracers in the familial form of AD. Recent Cryo-EM studies have demonstrated similar tau fibril folding structure in sAD and inherited AD (V717FAPP) [13] and it would be interesting to have similar studies comparing the tau folds in *AβPParc* and *PSEN1DE9*.

It should be born in mind that the different cutting levels in the large frozen brain sections for autoradiography and in the paraffin sections from the contralateral hemisphere used for immunostaining could have affected the comparison between the *AβPParc*, *PSEN1DE9*, and sAD brains as the same regions cannot be compared directly.

The results of the binding studies in brain homogenates were in general similar to those of the autoradiography studies on large frozen sections. The homogenization process of the brain most probably let us access to more binding sites than for the autoradiography. Indeed, on the autoradiography the tracer can reach only the accessible binding site, than when the brain is homogenized binding site that where inaccessible might become accessible. During the homogenization process, conformational and structural changes most probably occur in the cotton wool plaques and the ^3^H-PIB do not have access to the similar binding sites in comparison to amyloid plaques found in sAD brains. In silico computer modeling has suggested that there may be several binding sites on the amyloid fibril [17], as also suggested by in vitro binding with various amyloid ligands [18]. In this study, we observed the highest binding of ^3^H-PIB in the caudate nucleus of the *PSEN1DE9* brain in comparison with *AβPParc* and, despite the limitation of there being only one brain examined, this observation appears to be in agreement with in vitro results by Ni et al. [19] and in vivo results by Koivunen et al. [12]. The binding pattern for amyloid deposition is confirmed by binding assay as well as autoradiography and immunostaining. Binding of ^3^H-THK5117 and ^3^H-deprenyl in the entorhinal cortex of the *AβPParc2* brain was much higher than that in the *AβPParc1* brain and the sAD brain. When the results from the in vivo and in vitro studies were compared in *AβPParc1*, we observed a trend toward a negative association between ^18^F-FDG and ^3^H-THK5117 binding; no statistical analysis could be performed due to few data points. The time differences between in vivo/in vitro measurements might add some limitations. Increased tau deposits (as observed with ^3^H-THK5117) seemed to occur in regions with lower ^18^F-FDG PET data, implying lower cerebral metabolism and neuronal dysfunction.

There were several differences in amyloid plaque, tau deposition and activated astrocyte between the *AβPParc* and *PSEN1*D*E9* brains as well as in comparison with the sAD brains. A positive association was observed between activated astrocyte and tau loads for the *AβPParc* brains but no such correlation was observed, respectively, neither the *PSEN1*D*E9* nor the sAD brains. Although both mutations present abnormal plaque shapes (a ring shape for *AβPParc* and ‘cotton wool’ for *PSEN1*D*E9)*, these mutations in the *APP* and *PSEN1* genes, respectively, lead to major differences in the amyloid-beta composition and also in the tau deposits and activated astrocytes hallmarks in the development of AD pathology. Finally the use of two-tau tracers showed differences in regional binding both in the *PSEN1*D*E9* but also between the two *AβPParc* brains suggesting difference in AD variants, which deserves further exploration in vivo.

## Supplementary information

Supplementary data 1

Supplementary data 2

Supplementary data 3

## Data Availability

All data generated or analysed during this study are included in this published article.
