# Supplementary material for: Amyloid, tau, and astrocyte pathology in autosomal-dominant Alzheimer’s disease variants: AβPParc and PSEN1DE9
Source: Mol Psychiatry. 2020 Jun 25;26(10):5609–19. doi: 10.1038/s41380-020-0817-2 (PMC8758475; doi:10.1038/s41380-020-0817-2)
Supplement: Supplementary file 1 — Supplementary data 1 [file 41380_2020_817_MOESM1_ESM.docx]

Supplementary data 1

Clinical description of the patients with *AβPParc* and *PSEN1ΔE9* mutations

*AβPParc* mutation: Two descendants of a family from the north of Sweden (with a pedigree extending over five generations) who carried the *AβPParc* (p. E693G) mutation were included in the study [[2](#_ENREF_2)]. Mutation carrier ***AβPParc1*** started to experience memory problems at 53 years of age (mini-mental state examination [MMSE] score 28/30) and was diagnosed with Alzheimer’s disease at 62 years of age (MMSE score 23/30) followed by rapid cognitive decline (MMSE 11/30 at 64 years of age). The patient then participated in a multi-tracer PET study using ^11^C-PIB, ^11^C-L-deprenyl and ^18^F-FDG at 64 years of age, and died two years later, at age 66. The in vivo findings using ^11^C-PIB and ^18^F-FDG PET have been reported earlier by our group [[20](#_ENREF_20)]. Mutation carrier ***AβPParc2*** showed dysfunction in multiple cognitive areas at 61 years of age, followed by disorientation, dysphasia and anxiety, with rapid deterioration and death at 64 years of age after eights years of disease duration. A description of the pathology of ***AβPParc2*** (published as Sw2 in Kalimo et al., 2013) [[8](#_ENREF_8)].

*PSEN1ΔE9* mutation: The mutation carrier started to experience memory problems at 51 years of age and ^18^F-FDG PET exploration showed bi-temporal glucose hypometabolism [[25](#_ENREF_25)]. Eight years later (at 59 years of age) the subject showed severe dementia (MMSE 16/30) with spastic paraparesis, difficulties in swallowing, and speech dysarthria, but with preserved social ability and no psychiatric symptoms. ^11^C-PIB PET explorations at this stage of the disease showed especially a high uptake in the striatum [[9](#_ENREF_9)]. The patient died at 66 years of age.
