# Supplementary material for: Amyloid, tau, and astrocyte pathology in autosomal-dominant Alzheimer’s disease variants: AβPParc and PSEN1DE9
Source: Mol Psychiatry. 2020 Jun 25;26(10):5609–19. doi: 10.1038/s41380-020-0817-2 (PMC8758475; doi:10.1038/s41380-020-0817-2)
Supplement: Supplementary file 2 — Supplementary data 2 [file 41380_2020_817_MOESM2_ESM.docx]

Supplementary data 2


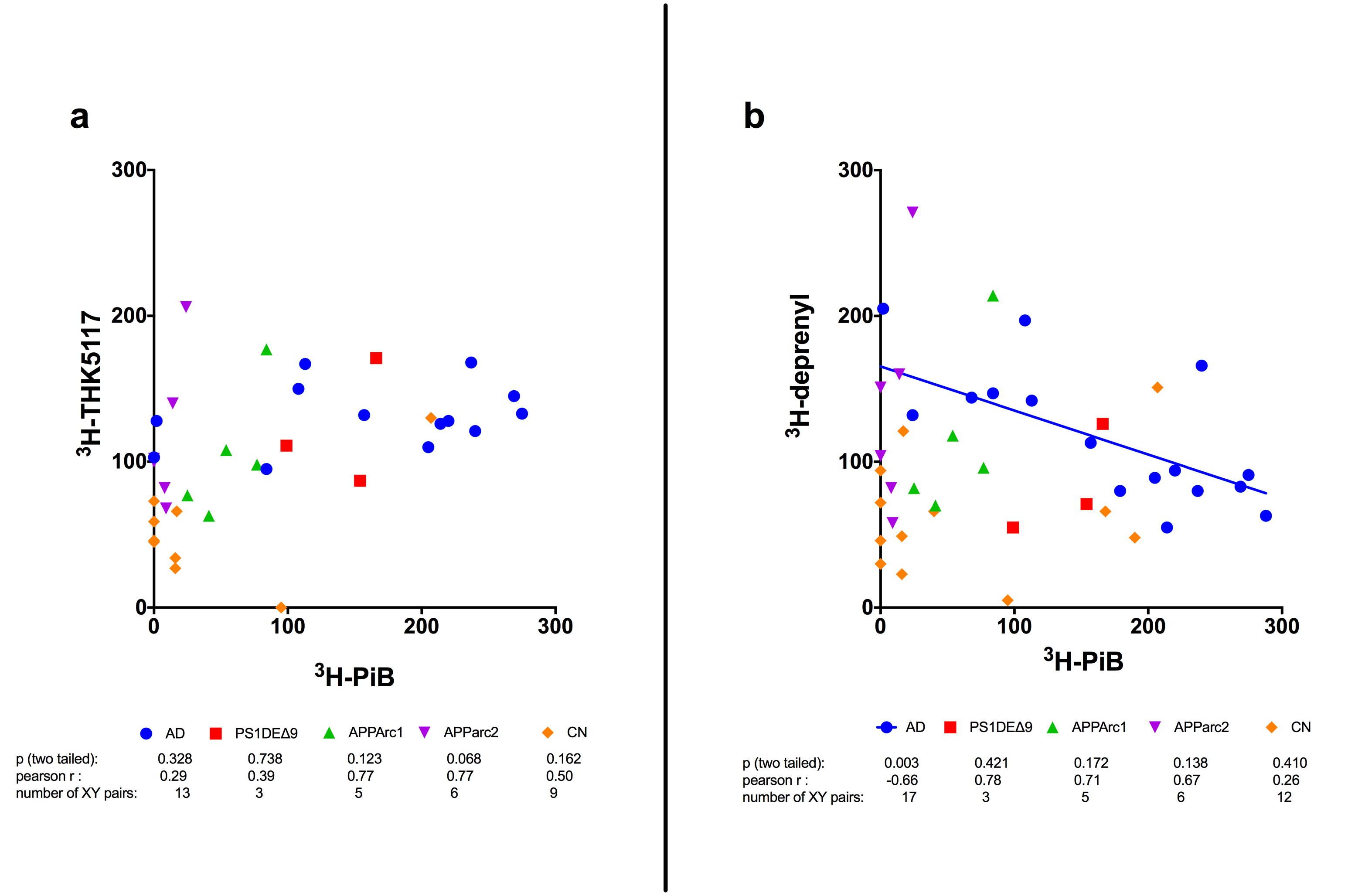


Comparison of ^3^H-deprenyl, ^3^H-THK5117 and ^3^H-PiB regional binding distributions

Correlation between ^3^H-THK5117 and ^3^H-PIB binding (a) and ^3^H-PIB and ^3^H-deprenyl (b) in two *AβPParc* brains, one *PSEN1*D*E9* brain, three Alzheimer’s disease brains and three normal control brains. Frontal cortex, temporal cortex, entorhinal cortex, caudate nucleus, and hippocampus tissue was used depending on availability. n= number of samples investigated.
