# Supplementary material for: Amyloid, tau, and astrocyte pathology in autosomal-dominant Alzheimer’s disease variants: AβPParc and PSEN1DE9
Source: Mol Psychiatry. 2020 Jun 25;26(10):5609–19. doi: 10.1038/s41380-020-0817-2 (PMC8758475; doi:10.1038/s41380-020-0817-2)
Supplement: Supplementary file 3 — Supplementary data 3 [file 41380_2020_817_MOESM3_ESM.docx]

Supplementary data 3:

In vitro-in vivo correlation:

In order to compare *in vitro* quantitative autoradiography binding measurements with *in vivo* quantification of the three PET tracers, the coronal section of the T1 MRI judged to correspond best to the relevant autoradiography section was manually segmented by two independent raters to define regions of interest, using the anatomical atlas used for segmentation of autoradiographies ^1^ and using MRIcron software^2^. For each of these regions, the mean uptake value of each PET tracer was sampled on the registered PET images. When agreement was satisfactory, regional uptake values from both raters were averaged for subsequent analyses. The relationship between regional uptake values measured by PET and the *in vitro* binding results from autoradiography was assessed using a Spearman correlation test.

Results

Correlation between PET scan and in vitro binding results in *AβPParc*1 brain

*AβPParc1* case was followed clinically and had undergone in vivo PET scan prior to death. We compared the in vivo binding of tracers with regional binding in vitro. Because THK5117 PET was not available at the time of in vivo exploration, we compared ^3^H-THK5117 in vitro binding to ^18^F-FDG PET measures.

A trend towards a negative association between ^18^F-FDG PET and ^3^H-THK5117 binding and positive relation between ^11^C-deprenyl binding and ^3^H-deprenyl binding across different brain regions could be observed which did not reach statistical significance. For each comparison, the number of regions for which both in vivo and in vitro measures were available was limited to four.


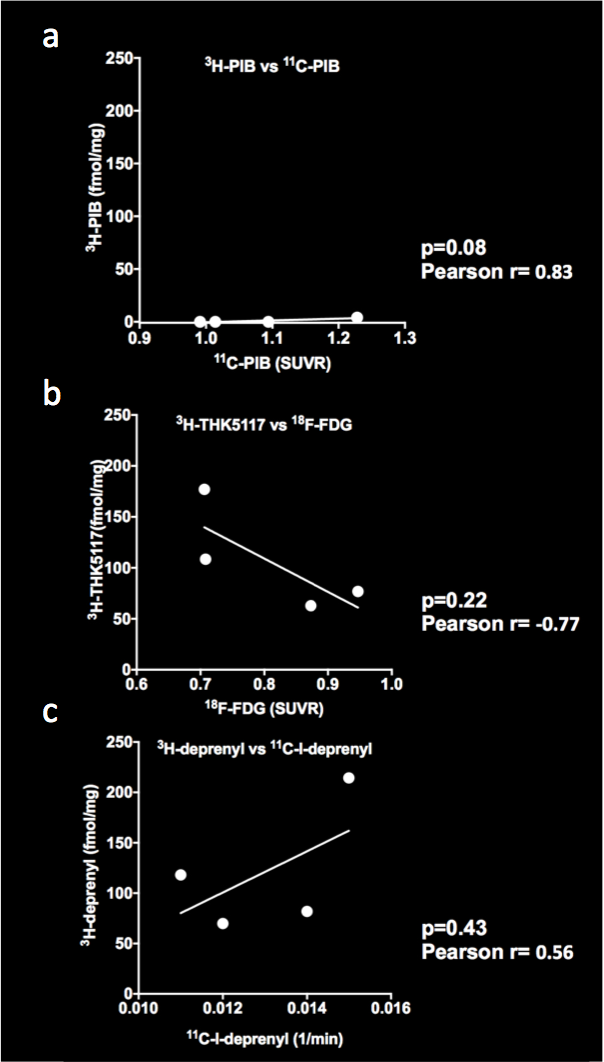


Associations between the in vitro binding results obtained from brain homogenate binding assays with ^3^H-PIB, ^3^H-THK5117, and ^3^H-deprenyl and the in vivo PET scan results with ^11^C-PIB, and ^18^F-FDG and ^11^C-l-deprenyl in an *AβPParc* mutation carrier (*AβPParc*1). a: ^3^H-PIB vs ^11^C-PIB, b: ^3^H-THK5117 vs ^18^F-FDG, c: ^3^H-deprenyl vs ^11^C-l-deprenyl

1. Naidich T. *Duvernoy’s Atlas of the Human Brain Stem and Cerebellum*, vol.

. Springer2009.

2. Rorden C, Brett M. Stereotaxic display of brain lesions. *Behav Neurol* 2000; **12**(4)**:** 191-200.
